# Surgical Outcomes of Lenvatinib Treatment Followed by Liver Resection for Advanced Hepatocellular Carcinoma Larger than 10 cm

**DOI:** 10.3390/cancers17172818

**Published:** 2025-08-28

**Authors:** Hideki Yokoo, Shoichiro Mizukami, Hiroyuki Takahashi, Tomoki Takizawa, Katsuro Enomoto, Kai Makino, Hiroki Takahata, Yuki Adachi, Koji Imai

**Affiliations:** Division of Hepato-Biliary-Pancreatic and Transplant Surgery, Department of Surgery, Asahikawa Medical University, Asahikawa 078-8510, Hokkaido, Japan; a102str@asahikawa-med.ac.jp (S.M.); takahiro@asahikawa-med.ac.jp (H.T.); ttomo0708@asahikawa-med.ac.jp (T.T.); k_enomoto@asahikawa-med.ac.jp (K.E.); makino-geka@asahikawa-med.ac.jp (K.M.); h-takahata@asahikawa-med.ac.jp (H.T.); a-yuki@asahikawa-med.ac.jp (Y.A.); kimai@asahikawa-med.ac.jp (K.I.)

**Keywords:** hepatocellular carcinoma, lenvatinib, conversion surgery, neoadjuvant therapy, large HCC, liver resection

## Abstract

Background: Hepatocellular carcinoma (HCC) larger than 10 cm has a poor prognosis, with high recurrence rates, particularly distant metastases. This study examined whether lenvatinib treatment followed by liver resection improves the outcomes for large HCCs compared with upfront surgery. Methods: We retrospectively analyzed 30 patients with HCC larger than 10 cm who underwent hepatectomy at our institution between January 2008 and December 2023. The study cohort included 30 patients: 9 received preoperative lenvatinib treatment followed by hepatectomy (LEN group), while 21 patients underwent upfront surgery (UFS group). We compared the clinicopathological characteristics, surgical outcomes, recurrence patterns, and survival between the two groups. Results: The median duration of lenvatinib administration was 1.8 months, with partial response in two patients (22.2%) and stable disease in seven patients (77.7%). While lenvatinib treatment significantly decreased serum albumin levels (*p* < 0.05) and increased ALBI scores (*p* = 0.03), the surgical outcomes including blood loss, operation time, and complication rates were comparable between the two groups. The 3-year recurrence-free survival rate was significantly higher in the LEN group compared with the UFS group (66.7% vs. 16.1%, *p* = 0.027). The 3-year overall survival rate was also higher in the LEN group, though not statistically significant (85.7% vs. 56.1%, *p* = 0.059). Notably, distant metastasis rates were lower in the LEN group compared with the UFS group (11.1% vs. 47.6%, *p* = 0.10). Conclusions: Preoperative lenvatinib treatment followed by hepatectomy for large HCC (> 10 cm) may reduce recurrence, particularly distant metastases, and potentially improve long-term survival. This approach may be a promising strategy for large HCCs, which traditionally have a poor prognosis with upfront surgery alone.

## Simple Summary

While surgical resection is recommended for early-stage hepatocellular carcinoma, the efficacy in patients with large hepatocellular carcinoma, greater than 10 cm, is only approximately 25–45%. Several studies have demonstrated improved outcome with surgical intervention after treatment with lenvatinib, a multikinase inhibitor, for advanced hepatocellular carcinoma. In this study, we investigated the potential efficacy of this strategy compared with surgery alone for patients with large hepatocellular carcinoma. The median follow-up period for the patient cohort (*n* = 30) was 30.1 months. The 3-year recurrence-free survival rate was significantly higher in the preoperative lenvatinib group (*n* = 9) compared with the surgery alone group (*n* = 21) (66.7% vs. 16.1%). The distant metastasis rate was significantly lower in the preoperative lenvatinib group compared with the surgery alone group (22.2% vs. 47.6%). These findings provide evidence for preoperative lenvatinib followed by hepatectomy as a potential therapeutic strategy for large hepatocellular carcinoma in reducing recurrence and improving patient survival.

## 1. Introduction

Primary liver cancer, which includes hepatocellular carcinoma (HCC), is one of the most common malignancies worldwide and the third leading cause of cancer-related deaths [1]. The management of HCC requires a multidisciplinary approach, with treatment strategies determined on the basis of tumor burden, liver function, and patient performance status. Clinical practice guidelines, including the Barcelona Clinic Liver Cancer staging system and the 5th edition of the Japan Society of Hepatology (JSH) HCC Guidelines, recommend surgical resection for early-stage HCC with preserved liver function [2,3]. However, the management of large HCCs, particularly those exceeding 10 cm in diameter, remains challenging.

Large HCC tumors are often associated with a poor prognosis as a result of their aggressive biological behavior and high recurrence rates [4,5]. Several studies have investigated the outcomes of surgical resection for large HCCs, with 5-year survival rates ranging from 25% to 45% [6,7,8]. A study from Hokkaido University in Japan reported that patients with huge HCC (> 10 cm) had significantly worse 5-year overall survival (OS) and recurrence-free survival (RFS) rates (42.9% and 14.2%, respectively) compared with patients with smaller tumors (71.3% and 33.1%, respectively) [5]. Furthermore, huge tumors were identified as an independent risk factor for initial extrahepatic recurrence, with a hazard ratio (HR) of 7.86 (*p* < 0.0001) [5]. The 5-year OS of patients with initial extrahepatic recurrence was significantly worse than that in patients with intrahepatic recurrence (16.8% vs. 50.5%) [5], highlighting the critical impact of distant metastases on long-term survival.

The concept of borderline resectable HCC (BR-HCC) has recently emerged as a new classification for HCCs that are technically resectable but oncologically problematic [9]. The BR-HCC Expert Consensus 2023 categorizes BR-HCC cases into two types: BR1 (technically resectable but with a high recurrence risk) and BR2 (technically challenging but potentially resectable), with both categories being recommended for preoperative neoadjuvant treatment [9]. These criteria effectively predicted surgical outcomes and survival rates in patients with advanced HCC [10]. Notably, in this classification, large HCCs could potentially fall into the resectable category. Given that research has demonstrated poor outcomes after surgery for these tumors, more effective strategies are needed for these patients [5,6,7,8].

Lenvatinib is a multikinase inhibitor targeting VEGFR1-3, FGFR1-4, PDGFRα, RET, and KIT that is used as a targeted treatment for cancer. In the REFLECT trial, lenvatinib treatment resulted in non-inferior survival outcomes compared with sorafenib treatment in the first-line treatment of unresectable HCC [11,12]. More recently, the LENS-HCC trial demonstrated high conversion surgical rates (67.3%) following lenvatinib treatment in patients with initially unresectable HCC, with promising 1-year OS rates of 75.9% [13]. Importantly, the trial showed that patients who underwent conversion surgery had better OS than those who did not undergo surgery, regardless of whether the initial unresectability was because of technical or oncological reasons. Several other studies have also reported favorable outcomes of surgical intervention after lenvatinib treatment for advanced HCC [14,15,16,17]. However, data on the efficacy of surgical intervention after lenvatinib treatment for large HCCs exceeding 10 cm in diameter are limited.

The mechanisms by which lenvatinib may improve outcomes after surgery include the induction of tumor shrinkage and significant tumor necrosis [18,19,20]. Lenvatinib inhibits tumor angiogenesis by targeting VEGF receptors and disrupts tumor cell proliferation by inhibiting fibroblast growth factor (FGF) receptors, leading to extensive tumor necrosis [21]. This necrotic effect may theoretically reduce the number of viable tumor cells that could disseminate into the systemic circulation during surgical manipulation, potentially decreasing the risk of distant metastases [22,23,24]. Lenvatinib has also been shown to have immunomodulatory effects, potentially enhancing anti-tumor immune responses [23,24].

We hypothesized that preoperative lenvatinib treatment may improve outcomes for patients with large HCC by reducing tumor burden, inducing tumor necrosis, and potentially controlling micrometastases before surgery. This study evaluated the surgical outcomes of patients with lenvatinib treatment followed by liver resection for large advanced HCC compared with those undergoing upfront surgical resection, with a particular focus on recurrence patterns and long-term survival.

## 2. Materials and Methods

### 2.1. Patient Population

We conducted a single-center retrospective analysis of patients who underwent hepatectomy for HCC larger than 10 cm at Asahikawa Medical University Hospital between January 2008 and December 2023. We collected clinical, laboratory, pathological, and surgical data for all enrolled patients. All patients underwent abdominal and chest computed tomography, abdominal magnetic resonance imaging, and ultrasound prior to surgery. Cases with negative distant metastasis using these modalities were candidates for hepatectomy. The Hokkaido University Algorithm incorporating the indocyanine green retention rate at 15 min and remnant liver volume was used to determine the surgical procedure, as described in previous report [25]. Patients were divided into two groups: those who received preoperative lenvatinib treatment followed by hepatectomy (LEN group) and those who underwent upfront surgery without preoperative systemic therapy (UFS group). This study was approved by the Ethics Committee of Asahikawa Medical University (No. 23010). The requirement for informed consent was waived because of the retrospective nature of the study.

### 2.2. Treatment Protocol

Since 2019, select patients with large HCC (> 10 cm) at our institution have been considered for preoperative lenvatinib treatment after multidisciplinary team discussions. The initial dose of lenvatinib is determined by body weight: 8 mg daily for patients weighing < 60 kg and 12 mg daily for those weighing ≥ 60 kg, with dose modifications based on tolerability [11]. Lenvatinib is administered for a minimum of 4 weeks, with radiological assessment performed every 4–8 weeks. Our lenvatinib treatment strategy was tailored to the presentations of individual patients with careful attention to preserving hepatic function for subsequent surgical intervention. For patients with technically unresectable disease, lenvatinib was administered until tumor shrinkage achieved resectability, with treatment continuation contingent upon maintaining a Child–Pugh score ≤ 6 points and modified albumin-bilirubin (mALBI) grade ≤ IIb. In patients with initially resectable disease, lenvatinib was used as neoadjuvant chemotherapy for approximately 2 months. Surgical procedures were performed following our institutional standards [25]. Anatomical resections were performed on the basis of Brisbane 2000 terminology [26]. The extent of hepatectomy was determined by tumor location, liver function reserve, and estimated future liver remnant volume. Toxicity was assessed following the Common Terminology Criteria for Adverse Events (CTCAE) version 5.0. The dose intensity was reduced by 4 mg if toxicity ≥ grade 3 was observed, and treatment was stopped if the patients no longer met the eligibility criteria.

### 2.3. Assessment Parameters

We collected the following data: patient demographics, viral status, tumor characteristics (size, number, vascular invasion), liver function parameters (Child–Pugh score, albumin-bilirubin [ALBI] grade, indocyanine green retention rate at 15 min [ICG R15]), and tumor markers (alpha-fetoprotein [AFP], des-gamma-carboxy prothrombin [DCP]).

For the LEN group, we analyzed lenvatinib dose intensity, duration of administration, response evaluation following the Response Evaluation Criteria in Solid Tumors (RECIST) version 1.1 and modified RECIST (mRECIST), and adverse events following CTCAE version 5.0. Changes in blood data before and after lenvatinib administration were also evaluated.

Surgical data included procedure type, blood loss, operation time, and postoperative complications. Pathological findings included tumor differentiation, gross type, vascular invasion, and TNM stage in accordance with the 8th edition of the Union for International Cancer Control classification. Tumor necrosis percentage was assessed in the resected specimens.

### 2.4. Outcome Measures

The primary outcome measures were RFS and OS. RFS was defined as the time from hepatectomy to the first evidence of recurrence or death. OS was defined as the time from hepatectomy to death from any cause or last follow-up. Secondary outcome measures included recurrence patterns (intrahepatic vs. extrahepatic) and surgical complications.

Patients were examined at 4-month intervals after surgery using blood tests and CT. If AFP or DCP values were elevated in the blood tests and recurrent tumors were apparent upon CT, we classified this as tumor recurrence. Dates of postoperative survival and recurrence were recorded and compared between the two groups.

### 2.5. Statistical Analysis

All statistical analyses were performed using EZR version 1.68 (Saitama Medical Center, Jichi Medical University, Saitama, Japan), a graphical user interface for R (The R Foundation for Statistical Computing, Vienna, Austria) and JMP version 14 for Windows (SAS Institute, Cary, NC, USA). Continuous variables were expressed as median with range and compared using the Mann–Whitney *U*-test. Categorical variables were expressed as numbers with percentages and compared using Fisher’s exact test. Survival curves were generated using the Kaplan–Meier method and compared using the log-rank test. A *p*-value < 0.05 was considered statistically significant.

## 3. Results

### 3.1. Patient Characteristics

A total of 30 patients with HCC larger than 10 cm who underwent hepatectomy were included in this study, with 9 patients in the LEN group and 21 patients in the UFS group. The baseline characteristics of both groups are summarized in Table 1. The detailed clinical characteristics of patients who received lenvatinib are summarized in Table S1. Regarding viral status, 9 patients (30%) were hepatitis B virus (HBV)-positive and 6 patients (20%) were hepatitis C virus (HCV)-positive, with no significant differences between groups (*p* = 0.924). Seven HBV-positive patients had received appropriate antiviral therapy with nucleos(t)ide analogues, with only one case achieving undetectable HBV DNA levels at the time of surgery. Among HCV-positive patients, 2 out of 4 (50%) had achieved a sustained virological response prior to surgery, with HCV RNA undetectable in these patients. Four HCV-positive patients had detectable HCV RNA at the time of surgery, but direct-acting antiviral therapy was initiated postoperatively. There were no significant differences in age, sex, viral status, tumor number, or tumor size between the two groups. However, the LEN group had significantly lower platelet counts compared with the UFS group (16.5 × 10^3^/µL vs. 25.4 × 10^3^/µL, *p* = 0.01). Tumor markers including AFP and DCP were comparable between the two groups.

Pathological examination revealed no significant differences in tumor differentiation, vascular invasion, or TNM stage between the two groups. There was a trend toward more poorly differentiated tumors (55.6% vs. 28.6%, *p* = 0.37) and more confluent multinodular type tumors in the LEN group (33.3% vs. 9.5%, *p* = 0.09). Notably, pathological examination of specimens from the LEN group often revealed extensive areas of tumor necrosis, particularly in the central regions of the tumors, consistent with the anti-angiogenic effects of lenvatinib.

### 3.2. Characteristics of Lenvatinib Treatment

In the LEN group, the median duration of lenvatinib administration was 1.8 months. Lenvatinib dosage was 4 mg daily in 3 patients, 8 mg daily in 4 patients, and 12 mg daily in 2 patients. Using the mRECIST criteria, 5 patients (55.6%) achieved partial response (PR) and 4 patients (44.4%) had stable disease (SD). When evaluated by RECIST criteria, which accounts for tumor necrosis, 2 patients (22.2%) achieved PR while 7 patients (77.7%) had SD. Among the responders according to mRECIST, 3 cases (60%) showed more than 70% tumor necrosis on pathological examination, demonstrating marked tumor-necrotizing effects of lenvatinib.

Grade 3 or higher adverse events were observed in one patient who developed intratumoral hemorrhage requiring transarterial embolization. Other adverse events were manageable with dose modifications (Table 2).

### 3.3. Changes in Liver Function After Lenvatinib Treatment

The comparison of laboratory data before and after lenvatinib administration revealed a significant decrease in serum albumin levels (median: 4.0 g/dL to 3.7 g/dL, *p* < 0.05) and a consequent increase in mALBI grade (*p* = 0.03) after lenvatinib treatment. The proportion of patients with modified ALBI grade IIb increased from 0% to 44.4% after lenvatinib treatment. There were no significant changes in platelet count, total bilirubin, ICG R15, or tumor markers after lenvatinib treatment (Table 3).

### 3.4. Surgical Outcomes

The surgical procedures, operative time, blood loss, and complication rates were comparable between the LEN and UFS groups (Table 4). Most patients in both groups underwent anatomical segmentectomy, sectionectomy, and hemihepatectomy. The median blood loss was 763 mL in the LEN group and 957 mL in the UFS group (*p* = 0.877). One patient in each group developed bile leakage as a postoperative complication.

### 3.5. Survival Outcomes and Recurrence Patterns

**Figure 1:**
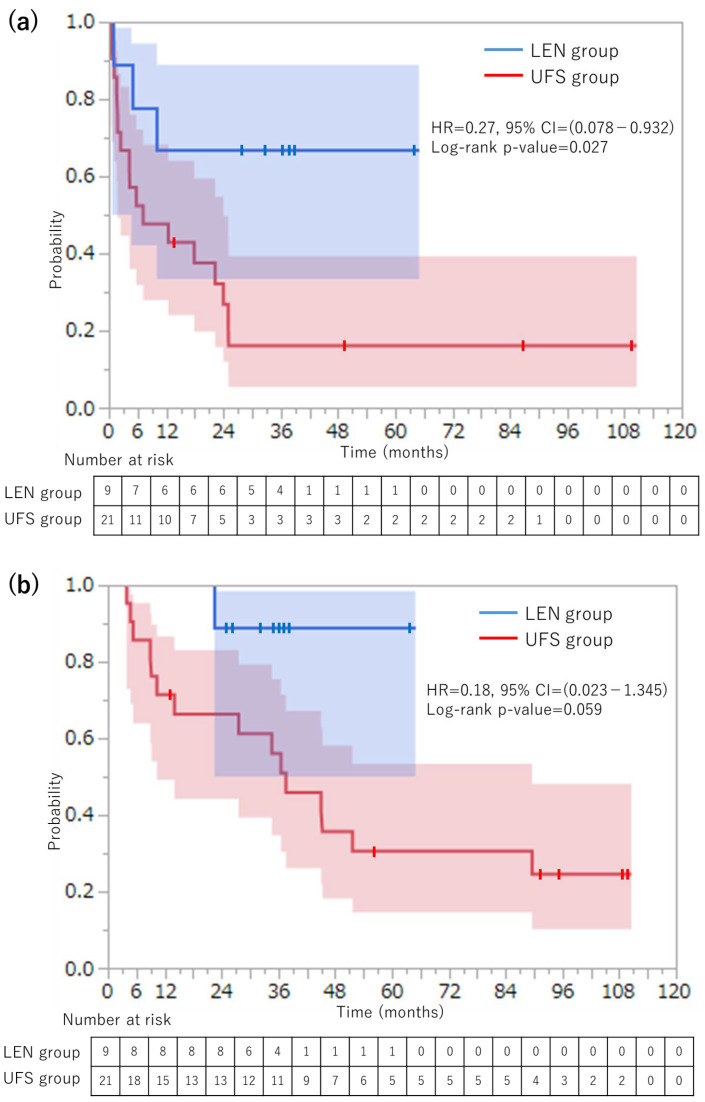
Kaplan–Meier survival curves comparing lenvatinib followed by hepatectomy (LEN) versus upfront surgery (UFS) for large hepatocellular carcinoma (> 10 cm). (**a**) Recurrence-free survival curves showing significantly better outcomes in the LEN group (*p* = 0.027). The 3-year recurrence-free survival rates were 66.7% for the LEN group and 16.1% for the UFS group. (**b**) Overall survival curves showing a trend toward better outcomes in the LEN group, though not statistically significant (*p* = 0.059). The 3-year overall survival rates were 85.7% for the LEN group and 56.1% for the UFS group. The blue and red shaded areas indicate the 95% confidence intervals. Survival analysis: log-rank test. (Blue and red shaded areas represent the 95% confidence intervals).

The median follow-up period was 30.1 months for the entire cohort. The 3-year RFS rate was significantly higher in the LEN group compared with the UFS group (66.7% vs. 16.1%, HR = 0.27, 95% confidence interval [CI]: 0.078–0.932, log-rank *p*-value = 0.027; Figure 1a). The 3-year OS rate was also higher in the LEN group, though not statistically significant (85.7% vs. 56.1%, HR = 0.18, 95% CI: 0.023–1.345, log-rank *p*-value = 0.059; Figure 1b). These results represent a notable improvement compared with historical data for large HCCs, where 5-year RFS rates of only 14.2% have been reported [5].

Analysis of recurrence patterns revealed that the distant metastasis rate was lower in the LEN group compared with the UFS group (22.2% [2/9] vs. 47.6% [10/21], *p* = 0.10). In the UFS group, common sites of distant metastasis included lung (5 patients), bone (5 patients), adrenal gland (1 patient), and lymph nodes (1 patient). In the LEN group, only 2 patients developed distant metastasis (adrenal gland and lung). Intrahepatic recurrence was observed in 3 patients in the LEN group and 10 patients in the UFS group.

## 4. Discussion

This study demonstrated that preoperative lenvatinib treatment followed by hepatectomy for large HCC (>10 cm) was associated with significantly improved RFS compared with upfront surgery. Moreover, preoperative lenvatinib administration may have contributed to a lower incidence of distant metastases. To the best of our knowledge, there have been no prior dedicated series focusing exclusively on this sequence in such large tumors. These findings suggest that this strategy could be a promising approach for large HCCs, which traditionally have poor prognosis with upfront surgery alone.

Large HCCs exceeding 10 cm have historically been associated with poor prognosis, primarily as a result of their aggressive biological behavior and high risk of recurrence, particularly extrahepatic metastases. Previous studies have reported 5-year survival rates of 25–45% for large HCCs treated with upfront surgery [5,6,7,8]. Our findings suggest that preoperative lenvatinib treatment may mitigate these risks, potentially by controlling micrometastases before surgery. This is supported by the markedly lower rate of distant metastasis observed in the LEN group compared with the UFS group (22.2% vs. 47.6%); this difference did not reach statistical significance, likely because of the small sample size.

The improved outcomes observed in our study may be attributed to several mechanisms of action of lenvatinib. First, lenvatinib induces significant tumor necrosis through its anti-angiogenic effects in targeting VEGF receptors [20,21]. This tumor necrosis effect is particularly relevant in the context of large HCCs, as it may reduce the number of viable tumor cells that could potentially disseminate into the systemic circulation during surgical manipulation. Pathological examination revealed significant tumor necrosis (>70%) in 60% of responders in the LEN group, which is consistent with previous reports on lenvatinib-induced tumor necrosis [20].

Second, lenvatinib inhibits the FGF signaling pathway, which plays a crucial role in tumor cell proliferation and metastasis [21]. By targeting multiple tyrosine kinase receptors, including FGFR1–4, lenvatinib may suppress the metastatic potential of tumor cells. Moreover, recent studies have suggested that FGF inhibition may also mitigate the immunosuppressive tumor microenvironment, potentially enhancing anti-tumor immune responses [23,24].

Third, the vascular normalization effect induced by anti-angiogenic agents such as lenvatinib may improve drug delivery to the tumor and enhance the efficacy of subsequent treatments [27]. This concept suggests that rather than completely destroying tumor vasculature, anti-angiogenic agents can transiently normalize the abnormal structure and function of tumor vessels, potentially improving tumor perfusion and drug delivery.

Our findings related to post-hepatectomy survival rates are particularly notable when compared with historical data on large HCCs. Previous studies have reported 3-year RFS rates of approximately 20–30% for large HCCs treated with upfront surgery [5,6,7,8], which is consistent with the 16.7% 3-year RFS rate observed in the UFS group. In contrast, the LEN group demonstrated a significantly higher 3-year RFS rate of 66.7% (*p* = 0.027), representing a substantial improvement in comparison with historical outcomes. Similarly, the 3-year OS rate in the LEN group (85.7%) was higher than previously reported rates for large HCCs [5,6,7,8], although the difference compared with the UFS group (56.1%) did not reach statistical significance (*p* = 0.059). The absence of statistical significance for the OS and the limited follow-up period necessitate cautious interpretation. Nevertheless, the present findings provide preliminary evidence that may inform future studies with larger cohorts and longer observation periods.

The efficacy of lenvatinib in our study is consistent with previous reports [28,29]. The LENS-HCC trial demonstrated high conversion rates (67.3%) with lenvatinib in patients with initially unresectable HCC [13]. Other studies have also reported favorable outcomes of surgical intervention after lenvatinib treatment for advanced HCC [14,15,16,17]. In our cohort, although tumor response according to RECIST criteria showed PR in only 22.2% of patients, pathological examination revealed marked tumor necrosis (>70%) in 60% of responders determined by mRECIST. This discrepancy highlights the anti-tumor effect of lenvatinib, which not only induces tumor shrinkage, but also significant necrosis [19,20].

One concern regarding preoperative lenvatinib treatment is its potential impact on liver function and surgical safety. We observed a significant decrease in serum albumin levels and an increase in ALBI grade after lenvatinib treatment. However, this did not translate into increased surgical complications or prolonged operative time, suggesting that the deterioration in liver function parameters was clinically manageable. These findings align with previous studies reporting the safety of conversion surgery after lenvatinib treatment [14,15,16,17].

From a pathological perspective, there were no significant differences in tumor characteristics between the two groups, although the LEN group tended to have more poorly differentiated tumors and confluent multinodular types. This might reflect a selection bias wherein patients with more aggressive tumor features were preferentially selected for preoperative lenvatinib treatment. Despite this potential bias, the LEN group demonstrated superior RFS, further supporting the efficacy of this approach.

The reduction in distant metastasis observed in our study is particularly significant given the high risk of extrahepatic recurrence associated with large HCCs. Wakayama et al. reported that huge HCC (>10 cm) was an independent risk factor for initial extrahepatic recurrence (HR 7.86, *p* < 0.0001) [5]. In our cohort, the distant metastasis rate was lower in the LEN group compared with the UFS group (22.2% vs. 47.6%, *p* = 0.10), suggesting that preoperative lenvatinib treatment may effectively control micrometastases that are undetectable by conventional imaging.

This potential suppression of distant metastasis may be explained by the tumor necrosis effect of lenvatinib, which may reduce the number of viable cancer cells disseminated into the systemic circulation during surgical manipulation. Recent studies have demonstrated the presence of circulating tumor cells (CTCs) in patients with HCC, with higher numbers of CTCs being associated with worse prognosis [22]. By inducing tumor necrosis preoperatively, lenvatinib may reduce the viability and number of CTCs released during surgery, thereby decreasing the risk of distant metastasis. Therefore, an important consideration in the management of advanced HCC following preoperative anti-VEGF therapy is the potential role of liver transplantation, which may indeed offer superior oncological outcomes compared with liver resection alone. The concept of ‘downstaging’ or ‘bridging’ therapy with lenvatinib might show promising results in rendering initially transplant-ineligible patients suitable for liver transplantation.

Our findings have important implications for the management of large HCCs. The recently proposed BR-HCC classification recognizes that certain HCCs, although technically resectable, have poor oncological outcomes with surgery alone [9,10]. Large HCC (>10 cm) is categorized as R-HCC; however, it has a high recurrence rate, particularly related to distant metastases. Our results provide evidence that preoperative lenvatinib treatment may be a valuable strategy for these borderline resectable tumors, potentially downstaging them oncologically before surgical intervention, as in the LENS-HCC trial. This aligns with the JSH HCC Guidelines’ emphasis on individualized treatment strategies. These findings may inform future guideline updates by providing evidence that preoperative lenvatinib can convert oncologically challenging large HCCs into more favorable surgical candidates, potentially shifting the treatment paradigm for this subset of patients.

The optimal duration of preoperative lenvatinib treatment remains controversial. In our cohort, the median duration of lenvatinib administration was 1.8 months, which is relatively short compared with the duration of neoadjuvant therapy in other cancer types. This protocol design was informed by evidence demonstrating that lenvatinib administration commonly causes a decline in hepatic function in the early treatment stage with a significant deterioration in ALBI scores from baseline after treatment initiation [30,31]. However, this relatively short duration was sufficient to achieve significant improvements in RFS. Further studies are needed to determine the optimal duration and regimen of preoperative lenvatinib treatment.

Whether combination therapy with immune checkpoint inhibitors could further enhance the efficacy of preoperative treatment is also worth exploring [32]. The combination of lenvatinib and pembrolizumab showed promising results in patients with unresectable HCC [33], and this combination may be more effective than lenvatinib monotherapy in the neoadjuvant setting. However, the safety and efficacy of this combination as preoperative therapy for resectable HCC require further investigation.

This study has several limitations. First, this was a retrospective analysis with a small sample size, which limits the statistical power and increases the risk of type II errors. Indeed, in the post hoc power analysis of survival and distant metastasis rates, the statistical power was 68.4% and 23.3%, respectively, both of which fell short of the 80% benchmark, indicating a limitation related to insufficient sample size. Second, there was a potential selection bias, as the decision to administer preoperative lenvatinib was determined by multidisciplinary team discussions rather than randomized allocation. Third, the follow-up period was relatively short, and a longer follow-up is needed to confirm the sustained benefit on OS. Fourth, as this study was performed in a single-center setting and limited to patients with large (≥10 cm) HCC, the external validity may be constrained. To enhance the generalizability of these findings, future multicenter studies with larger patient cohorts are warranted.

## 5. Conclusions

Our study suggests that preoperative lenvatinib treatment followed by hepatectomy for large HCC (>10 cm) may reduce recurrence rates, particularly distant metastases, and potentially improve long-term survival compared with upfront surgery. However, the preliminary nature of our overall survival findings requires confirmation in larger, prospective studies with extended follow-up periods. This approach appears safe and feasible, without increasing surgical complications, despite mild deterioration in liver function parameters. The significant improvement in RFS and the trend toward reduced distant metastases support the hypothesis that the tumor necrosis effect of lenvatinib may suppress viable cancer cell dissemination into the systemic circulation. These findings suggest that preoperative lenvatinib may be a valuable treatment strategy for large HCCs, which have traditionally been associated with poor outcomes following upfront surgery alone. Prospective, multicenter studies with larger sample sizes are needed to validate these results and establish the optimal duration and regimen of preoperative lenvatinib treatment.

## Figures and Tables

**Table 1:** Baseline characteristics of patients with hepatocellular carcinoma treated with upfront surgery or conversion surgery after lenvatinib.

Characteristics	UFS (*n* = 21)	LEN-S (*n* = 9)	*p*-Value
Age, years, median (range)	69 (32–79)	75 (52–77)	0.468
Male sex, *n* (%)	16 (76.2)	8 (88.9)	0.426
Virus, *n* (%)			
HBV	6 (28.6)	3 (33.3)	
HCV	4 (19.1)	2 (22.2)	0.924
NBNC	11 (52.4)	4 (44.4)	
Previous antiviral therapy, *n* (%)			
HBV patients treated with nucleos(t)ide analogues	4/6 (66.7)	3/3 (100)	0.257
HCV patients achieved SVR	2/4 (50.0)	0/0 (0)	-
Viral status at resection, *n* (%)			
HBV DNA undetectable	0/6 (0)	1/3 (33.3)	0.134
HCV RNA undetectable	2/4 (50.0)	0/2 (0)	0.221
Platelet count ×10^4^/µL, median (range)	25.4 (7–39.4)	16.5 (13.5–34)	0.004
Child–Pugh score, *n* (%)			
5	19 (90.5)	9 (100)	0.338
6	2 (9.5)	0 (0)	
Albumin, g/dL, median (range)	3.7 (2.5–4.5)	4.0 (3.6–4.4)	0.803
T-Bil, mg/dL, median (range)	0.8 (0.3–1.0)	0.9 (0.6–1.8)	0.734
mALBI grade, *n* (%)			
I	7 (33.3)	4 (44.4)	
IIa	9 (42.9)	5 (55.6)	0.276
IIb	5 (23.8)	0 (0)	
Indocyanine green retention rate at 15 min %, median (range)	9.7 (3.5–33.4)	13.8 (6–25.2)	0.192
AFP, ng/mL, median (range)	50.2 (3–200,522)	201 (3–259,868)	0.803
DCP, mAU/mL, median (range)	3237 (14–101,790)	1642 (178–52,856)	0.734
Tumor number, *n* (%)			
Single	14 (66.7)	7 (77.8)	0.543
Multiple	7 (33.3)	2 (22.2)	
Gross type, *n* (%)			
SN	6 (28.6)	0 (0)	
SNEG	13 (61.9)	6 (66.7)	0.09
CMN	2 (9.5)	3 (33.3)	
Tumor differentiation, *n* (%)			
Well	2 (9.5)	0 (0)	
Moderate	13 (61.9)	4 (44.4)	0.294
Poor	6 (28.6)	5 (55.6)	
Portal vein invasion, *n* (%)	10 (47.6)	5 (55.6)	0.69
Hepatic vein invasion, *n* (%)	9 (42.9)	3 (33.3)	0.626
TNM stage, *n* (%)			
IA	0 (0)	0 (0)	
IB	6 (28.6)	3 (33.3)	
II	6 (28.6)	4 (44.4)	0.592
IIIA	6 (28.6)	2 (22.2)	
IIIB	3 (14.2)	0 (0)	

Abbreviations: UFS, upfront surgery; LEN-S, conversion surgery after lenvatinib; HBV, hepatitis B virus; HCV, hepatitis C virus; NBNC, non-B non-C; T-Bil, total bilirubin; mALBI, modified albumin-bilirubin; AFP, alpha-fetoprotein; DCP, des-gamma-carboxy prothrombin; SN, simple nodular type; SNEG, simple nodular type with extranodular growth; CMN, confluent multinodular type; SVR, sustained virological response; TNM, Tumor, Node, Metastasis. Continuous variables: Mann–Whitney *U*-test, categorical variables with expected frequencies ≥5: Chi-squared test, categorical variables with expected frequencies <5: Fisher’s exact test.

**Table 2:** Background of lenvatinib administration in the lenvatinib group.

Characteristics	
Efficacy of lenvatinib based on RECIST, *n* (%)	
Complete response	0 (0)
Partial response	2 (22.2)
Stable disease	7 (77.8)
Progressive disease	0 (0)
Efficacy of lenvatinib based on mRECIST, *n* (%)	
Complete response	0 (0)
Partial response	5 (55.6)
Stable disease	4 (44.4)
Progressive disease	0 (0)
Duration of administration in days, median (range)	72 (24–184)
Dose of intensity, *n* (%)	
4 mg	3 (33.3)
8 mg	4 (44.4)
12 mg	2 (22.3)
Adverse event, *n* (%)	
Hemorrhage	1 (11.1)
General fatigue	1 (11.1)

Abbreviations: RECIST, Response Evaluation Criteria in Solid Tumors; mRECIST, modified Response Evaluation Criteria in Solid Tumors.

**Table 3:** Changes in data before and after 2 months of lenvatinib administration.

Parameters	Before	After	*p*-Value
Platelet count ×10^4^/µL, median (range)	16.5 (13.5–34)	15.7 (11.1–32.1)	0.507
Albumin in mg/mL, median (range)	4 (3.6–4.4)	3.7 (2.8–4.2)	0.045
Total bilirubin in mg/mL, median (range)	0.9 (0.6–1.8)	0.7 (0.5–1.9)	0.248
mALBI grade, *n* (%)			
I	4 (44.4)	3 (33.3)	
IIa	5 (55.6)	2 (22.2)	0.03
IIb	0 (0)	4 (44.4)	
Child–Pugh score, *n* (%)			
5	9 (100)	6 (66.7)	0.03
6	0 (0)	3 (33.3)	
Indocyanine green retention rate at 15 min %, median (range)	13.8 (6–25.2)	13.2 (7.8–37.6)	0.658
AFP in ng/mL, median (range)	201 (3–259,868)	33.5 (2–59,912)	0.48
DCP in mAU/mL, median (range)	1642 (178–52,856)	1430 (37–106,829)	0.691

Abbreviations: mALBI, modified albumin-bilirubin; AFP, alpha-fetoprotein; DCP, des-gamma-carboxy prothrombin. Continuous variables: Mann–Whitney *U*-test, categorical variables with expected frequencies ≥5: Chi-squared test, categorical variables with expected frequencies <5: Fisher’s exact test.

**Table 4:** Surgical outcomes of patients with hepatocellular carcinoma treated with upfront surgery or conversion surgery after lenvatinib.

Outcomes	UFS (*n* = 21)	LEN-S (*n* = 9)	*p*-Value
Surgical procedures, *n* (%)			
Segmentectomy, sectionectomy	10 (47.6)	3 (33.3)	0.636
Central hepatectomy or Hemihepatectomy	11 (52.4)	6 (66.7)	
Operation time, min, median (range)	459.5 (190–707)	478 (352–1000)	0.643
Blood loss, mL, median (range)	956.5 (73–6453)	763 (223–12,601)	0.877
Morbidity, *n* (%)			
Bile leakage	1 (4.8)	1 (11.1)	0.522
Postoperative hospital stay, days, median (range)	19 (5–69)	10 (8–95)	0.186
90-day mortality, *n* (%)	0 (0)	0 (0)	

Abbreviations: UFS, upfront surgery; LEN-S, conversion surgery after lenvatinib. Continuous variables: Mann–Whitney *U*-test, categorical variables with expected frequencies ≥5: Chi-squared test, categorical variables with expected frequencies <5: Fisher’s exact test.

## Data Availability

The datasets presented in this article are not readily available because of privacy and ethical restrictions.

## Supplementary Materials

The following supporting information can be downloaded at: https://www.mdpi.com/article/10.3390/cancers17172818/s1, Table S1: [Clinical Characteristics of Patients in the LEN Group].

## Abbreviations

The following abbreviations are used in this manuscript:

HCC	Hepatocellular carcinoma
OS	Overall survival
RFS	Recurrence-free survival
BR-HCC	Borderline resectable HCC
CTCAE	Common Terminology Criteria for Adverse Events
ALBI	Albumin-bilirubin
ICG R15	Indocyanine green retention rate at 15 min
AFP	Alpha-fetoprotein
DCP	Des-gamma-carboxy prothrombin
RECIST	Response Evaluation Criteria in Solid Tumors
mRECIST	Modified RECIST
PR	Partial response
SD	Stable disease
